# Adult presentation of cor triatriatum sinister with anomalous coronary venous drainage into the left atrium: A rare congenital abnormality

**DOI:** 10.1016/j.radcr.2026.06.098

**Published:** 2026-07-18

**Authors:** Nile Luu, Randy Richardson

**Affiliations:** a School of Medicine, Creighton University School of Medicine, Phoenix, AZ, USA; b Department of Radiology, Creighton University School of Medicine, Phoenix, AZ, USA

**Keywords:** Cor triatriatum sinister, Anomalous coronary venous drainage, Case report, Computed tomography imaging, Congenital heart disease

## Abstract

Cor triatriatum is a rare congenital heart defect characterized by an abnormal fibromuscular septation. Many cases present in infancy, with adult presentations especially uncommon. Additionally, concomitant anomalous coronary venous drainage into the left atrium is extremely rare, with few cases reported in the literature. We report the case of a 71 year old male with progressive dyspnea on exertion and severe symptomatic aortic valve stenosis who was found on CT angiography to have evidence of an incomplete fibromuscular membrane within the left atrium consistent with cor triatriatum sinister, as well as anomalous coronary venous drainage with the coronary sinus draining into the left atrium. This case highlights the importance of advanced cardiac imaging in discovering rare congenital cardiac anomalies in adults with cardiovascular symptoms as it can aid in diagnostic clarification, procedural planning and cardiovascular interventions. Recognition of these abnormalities is particularly important in patients undergoing structural cardiac evaluation, as there may be implications for procedural planning or management.

## Introduction

Cor triatriatum is a rare congenital heart defect characterized by an abnormal fibromuscular septation in the right (cor triatriatum dexter [CTD]) or left (cor triatriatum sinister [CTS]) atrium that divides the corresponding atrium into separate chambers. CTS is the most common abnormality between the two, with CTS appearing in an estimated 0.4% of congenital heart defects and CTD having an estimated incidence of 0.025% [[1], [2], [3]]. Cor triatriatum commonly presents as an isolated defect or with other abnormalities such as persistent levoatrial cardinal veins, atrial or ventricular septal defect, or other heart defects [[4], [5], [6]]. Additionally, patients with cor triatriatum present with a wide spectrum of clinical manifestations, ranging from asymptomatic disease to dyspnea or symptoms resembling mitral stenosis [7].

Similarly, anomalies of the coronary sinus and coronary venous drainage are rare and may coexist with other complex congenital cardiac malformations. Abnormalities such as coronary sinus malformations and anomalous coronary venous drainage to sites other than the right atrium have an estimated prevalence of 0.5% of congenital heart defects and may present asymptomatically or with signs of right to left shunting [[8], [9], [10]]. In this report, we describe a patient with cor triatriatum sinister with associated anomalous coronary venous drainage into the left atria. To our knowledge, the presence of these two anomalies has rarely been reported in the literature. Recognition of these abnormalities may be clinically significant for adults undergoing structural cardiac evaluation as it may aid in procedural planning or interventions.

## Case presentation

A 71 year old male with a past medical history of atrial fibrillation, mild nonobstructive coronary artery disease, hypercholesteremia, prediabetes, no smoking history, and severe symptomatic aortic valve stenosis with an ejection fraction of 60%, a valve area 0.8 cm², a mean gradient of 40 mmHg, and peak velocity of 4.5 m/s, presented with progressively worsening dyspnea on exertion for evaluation for transcatheter valve replacement (TAVR). He denied chest pain, syncope, palpitations, however endorsed activity fatigue and dyspnea. He had a history of radiofrequency ablation for atrial fibrillation and the placement of a permanent pacemaker. Family history was noncontributory for congenital or premature cardiovascular disease. Patient medications included aspirin, apixaban, and pravastatin. On a physical exam, blood pressure was 144 / 85 mmHg, heart rate was 76 beats per minute, and oxygen saturation was 95% on room air. There was additionally presence of a systolic murmur on auscultation, with the remainder of the exam within normal limits. Routine laboratory evaluation was not contributory.

Due to the symptomatic aortic stenosis, the patient underwent a chest CT angiogram for pre-procedural evaluation for TAVR. On TAVR CTA, a bicuspid aortic valve was identified, along with a 4.3 cm ascending aortic aneurysm and a 4.4 cm sinus of Valsalva aneurysm involving the noncoronary sinus. Most notably, CTA imaging demonstrated an incomplete membranous band in the left atrium along the interatrial septum/fossa ovalis, consistent with cor triatriatum sinister (Fig. 1). Anomalous coronary venous drainage additionally was identified with the left ventricular vein, great cardiac vein, middle cardiac vein, and small cardiac vein draining into the coronary sinus, which demonstrated drainage into the left atrium rather than the right atrium (Fig. 2). Three dimensional volume rendered reconstructions further delineated the anomalous venous anatomy and the adjacent cardiac structures (Fig. 3).

**Fig. 1:**
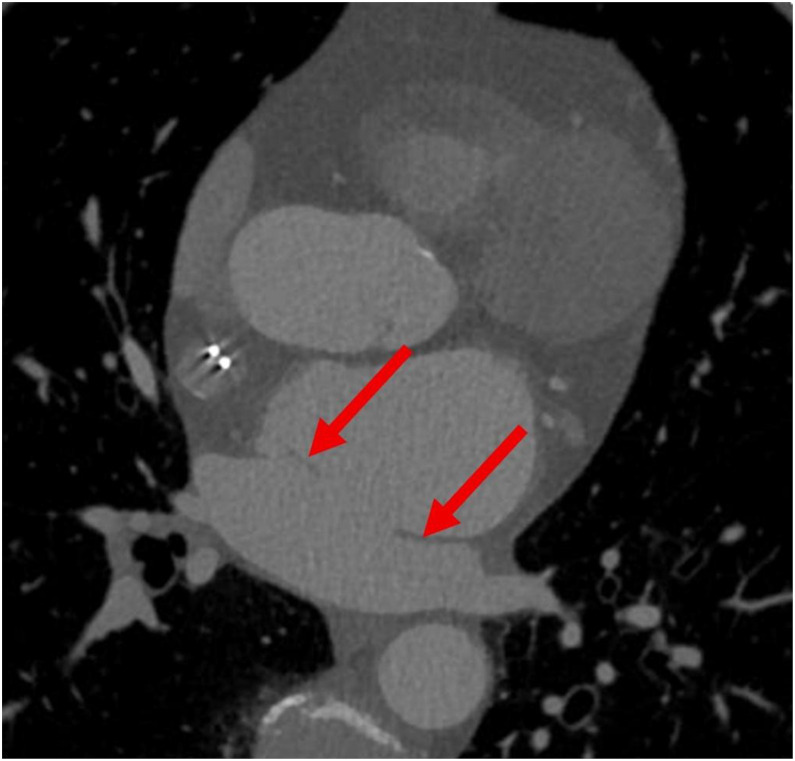
Axial computed tomography image of the fibrous membrane within the left atrium (red arrow).

**Fig. 2:**
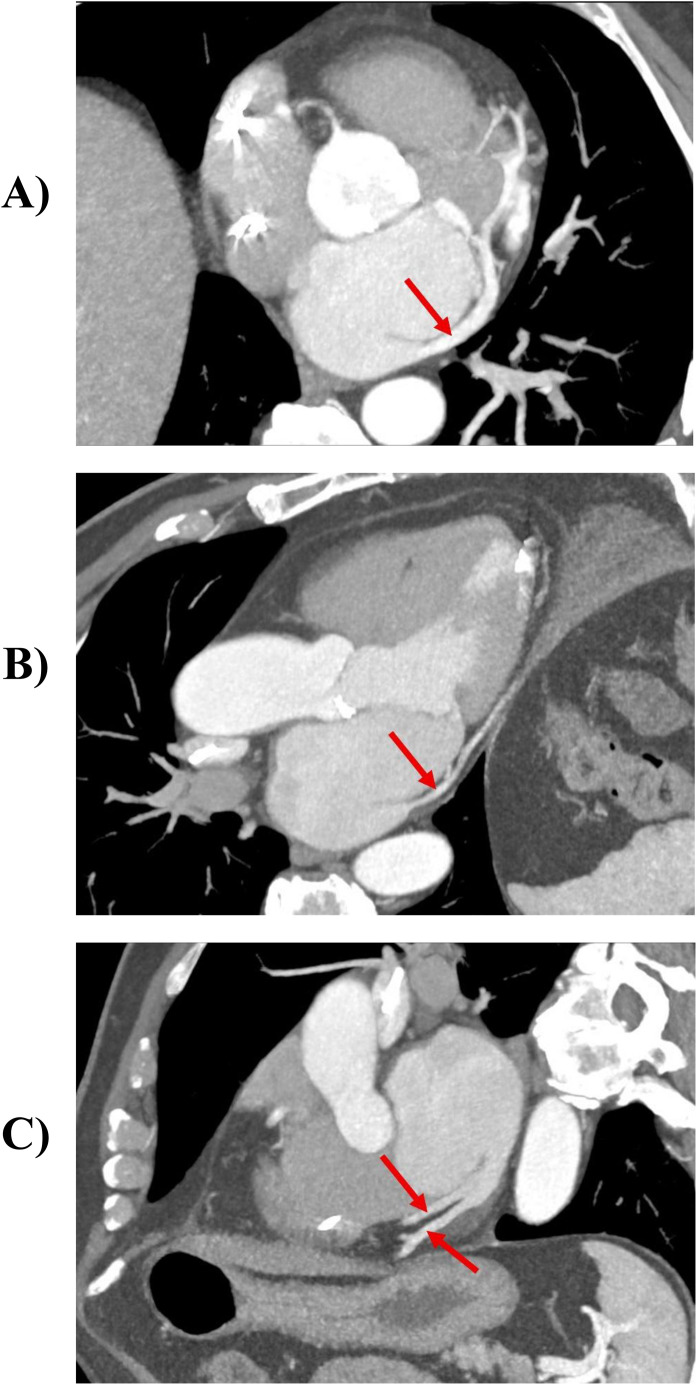
Axial computed tomography images demonstrating abnormal coronary venous drainage of the great cardiac vein (A), left ventricular vein (B), and small and middle cardiac veins (C) into the left atrium with red arrows labeling each structure.

**Fig. 3:**
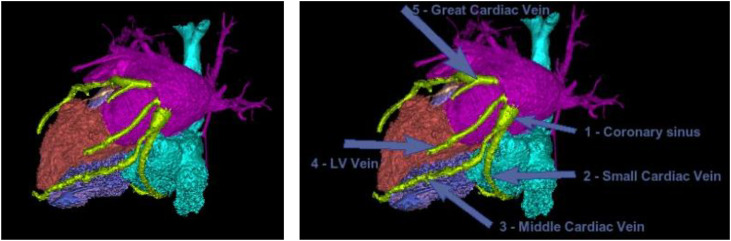
Three-dimensional reconstruction of a computed tomography image demonstrating abnormal coronary venous drainage into the left atrium with blue arrows labeling each structure.

Following evaluation, the patient underwent successful transcatheter aortic valve replacement with implantation of a bioprosthetic aortic valve. Post procedural transthoracic echocardiography demonstrated appropriate prosthetic valve function without evidence of procedural complications. The examination also noted suspected cor triatriatum sinister without evidence of pulmonary venous flow restriction. At a 30 day follow up, the patient remained clinically stable and without concerns.

## Discussion

This case presents a rare combination of CTS with anomalous coronary venous drainage that presented with severe aortic stenosis. Although cor triatriatum is typically diagnosed in childhood, adult cases are often detected incidentally during imaging performed for unrelated cardiovascular disease.

The etiology of CTS likely involves the incomplete incorporation of the common pulmonary vein into the left atrium during embryogenesis, resulting in the presence of a fibromuscular membrane. Other theories suggest that the membrane arises from an abnormal growth of the septum primum [11]. Regardless, persistence of the fibromuscular membrane leads to partitioning of the left atrium into multiple chambers, with the proximal chamber receiving pulmonary venous flow, while a distal chamber contains the mitral valve and other cardiac anatomy. When identified in adults, clinical manifestations may present variably and may be dependent on membrane morphology and fenestration size. Those with restrictive membranes can present with more severe symptoms such as progressive dyspnea, pulmonary hypertension, or right ventricular failure, while nonrestrictive membranes may present asymptomatically [12]. In this patient, follow up echocardiography demonstrated suspected cor triatriatum sinister without evidence of pulmonary venous flow restriction, suggesting that the membrane likely was nonobstructive. The absence of significant obstruction may explain the patients delayed diagnosis at an older age.

In contrast, anomalous coronary venous drainage reflects aberrant development of the sinus venosus, leading to drainage of the coronary veins to sites outside of the right atrium. There is a spectrum of coronary venous abnormalities ranging from unroofed coronary sinus syndrome, persistent left superior vena cava, hypoplasia or absence of the coronary sinus and other rare drainage patterns, reflecting disruption of normal coronary sinus formation [10].

Although rare, cor triatriatum sinister has been commonly associated with other cardiac abnormalities such as atrial or ventricular septal defects and persistent left superior vena cava, suggesting that cor triatriatum may be related to broader spectrum of venous or atrial development [13]. Similarly, coronary venous anomalies are frequently associated with systemic or pulmonary venous malformations, suggesting a possible overlap in developmental pathways affecting atrial inflow structures.

Cases of cor triatriatum with concomitant anomalous coronary sinus drainage into the left atrium are exceedingly rare and may be underreported in the literature. Although both cor triatriatum sinister and coronary venous abnormalities arise from distinct embryological pathways, both reflect abnormalities involving atrial inflow tract formation, with the pulmonary venous system involved in CTS development and sinus venous remodeling involved in coronary sinus development. Therefore, the coexistence of these defects suggests a possible connection in embryologic development, rather than entirely independent anomalies.

In this patient, the cause of his exertional dyspnea may have been multifactorial, reflecting obstruction from aortic stenosis as well as possibly altered left atrial hemodynamics due to the presence of cor triatriatum, right to left shunting, or the altered flow from the coronary venous anomalies. From a clinical standpoint, recognition of such rare coexisting congenital anomalies is important in adult patients undergoing structural cardiac evaluation, as it may alter surgical planning.

## Conclusions

Adult presentation of CTS with anomalous coronary venous drainage is rare. This case underscores the importance of detailed imaging in detecting cardiac abnormalities in patients with cardiovascular symptoms. Recognition and detection of these coronary venous anomalies and cor triatriatum with imaging is particularly critical for surgical planning in cases that require intervention to prevent injury to these venous structures.

## Patient consent

Informed written consent for publication was obtained or waived by all participants the patient in this study.
